# Conformational rigidity of silicon-stereogenic silanes in asymmetric catalysis: A comparative study

**DOI:** 10.1186/1860-5397-3-9

**Published:** 2007-02-08

**Authors:** Sebastian Rendler, Martin Oestreich

**Affiliations:** 1 Organisch-Chemisches Institut, Westfälische Wilhelms-Universität Münster, Corrensstrasse 40, D-48149 Münster, Germany

## Abstract

In recent years, cyclic silicon-stereogenic silanes were successfully employed as stereoinducers in transition metal-catalyzed asymmetric transformations as exemplified by (1) the hydrosilylation of alkenes constituting a chirality transfer from silicon to carbon and (2) the kinetic resolution of racemic mixtures of alcohols by dehydrogenative silicon-oxygen coupling. In this investigation, a cyclic and a structurally related acyclic silane with silicon-centered chirality were compared using the above-mentioned model reactions. The stereochemical outcome of these pairs of reactions was correlated with and rationalized by the current mechanistic pictures. An acyclic silicon-stereogenic silane is also capable of inducing excellent chirality transfer (*ct*) in a palladium-catalyzed intermolecular carbon-silicon bond formation yet silicon incorporated into a cyclic framework is required in the copper-catalyzed silicon-oxygen bond forming reaction.

## Findings

Within the last decade, several asymmetric transformations based on silicon-stereogenic reagents or substrates were revisited or invented. [1–4] Aside from the use of silicon-stereogenic chiral auxiliaries in substrate-controlled reactions, [5] a still limited number of remarkable stereoselective processes with a stereogenic silicon as the reactive site were reported, [6] namely the inter- [7] as well as intramolecular [8] chirality transfers from silicon to carbon. Moreover, we had demonstrated that chiral silanes resolve racemic mixtures of alcohols in a non-enzymatic, transition metal-catalyzed kinetic resolution. [9]

**Figure 1:**
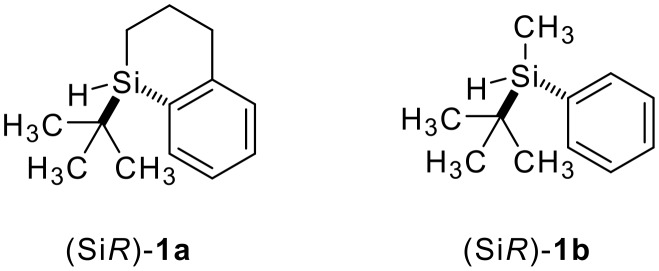
Cyclic and acyclic sterically encumbered silanes.

During our ongoing investigations directed towards the mechanistic elucidation of the origin of the chirality transfer in a palladium-catalyzed hydrosilylation, [10] we had to perform an extensive screening of silicon-stereogenic tertiary silanes. On that occasion, we became aware that a similar level of stereoselection was obtained when priveleged cyclic system **1a** [11] was exchanged for the important acyclic congener **1b** [12–15] (Figure 1). We had erroneously missed this known tertiary silane. This was particularly unfortunate in the light of the fact that these silanes are both decorated with three substituents of different steric demand and, therefore, display marked stereochemical differentiation around silicon.

In this preliminary communication, we wish to report a comparison of cyclic **1a** and acyclic **1b** as stereoinducers in the palladium-catalyzed chirality transfer from silicon to carbon and in the copper-catalyzed kinetic resolution of donor-functionalized alcohols capable of two-point binding.

**Scheme 1:**
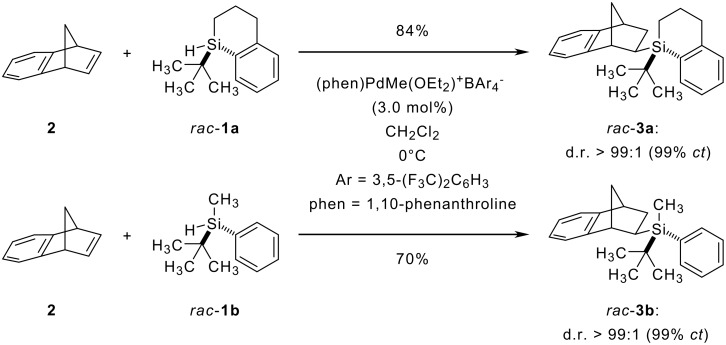
Cyclic and acyclic chiral silanes as potent reagents for the silicon-to-carbon chirality transfer.

The reagent-controlled hydrosilylation of norbornene derivative **2** with silane **1a** proceeds with a perfect chirality transfer (*rac*-**1a** → *rac*-**3a**, Scheme 1). [8] Mechanistic investigation of the nature of the stereochemistry-determining step in this catalysis required a silane, which would produce slightly diminished diastereoselectivity and, hence, attenuated chirality transfer from silicon to carbon. [10] It was that situation that prompted us to investigate a considerable range of silicon-stereogenic silanes initially varied in ring size and exocyclic substituent; this was not met with satisfactory success. Based on the assumption that less rigid acyclic silanes would induce lower levels of diastereoselection, previously reported silane *rac*-**1b** – readily prepared in its racemic form [13] – was then supposed to serve such purpose. To our surprise, the palladium-catalyzed hydrosilylation of **2** with *rac*-**1b** gave almost perfect diastereoselectivity and good yield (*rac*-**1b** → *rac*-**3b**, Scheme 1).

**Scheme 2:**
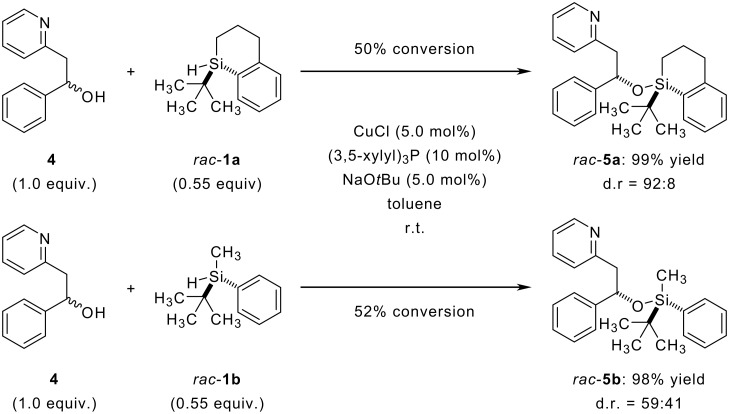
Kinetic resolution of secondary alcohols using a dehydrogenative coupling reaction.

This unexpected result inevitably introduced the pivotal question whether conformational rigidity of chiral silanes is a dispensible characteristic for asymmetric transformations. Thus, we subsequently tested *rac*-**1b** as resolving reagent in the kinetic resolution of an alcohol with a pending nitrogen donor (Scheme 2). In an earlier report, enantiomerically enriched silane **1a** (96% *ee*) was applied in this diastereoselective copper-catalyzed dehydrogenative silicon-oxygen coupling affording promising optical purities for the unreacted alcohol *ent*-**4** (84% *ee*) along with **5** (d.r. = 84:16) at 56% conversion. [9] For the present study, the diastereoselectivity of the formed ethers **5** is conclusive, which, in turn, allows for working with racemic silanes *rac*-**1** (*rac*-**1a** → *rac*-**5a** versus *rac*-**1b** → *rac*-**5b**, Scheme 2). This is sufficient since the d.r. of **5** will be identical to the e.r. of the remaining alcohol **4** at exactly 50% conversion when using enantiopure silane **1**. It must be noted that that diastereoselectivity is not dependent on conversion when using racemic silanes *rac*-**1**; conversely, using enantioenriched **1** it is.

Whereas *rac*-**5a** was formed highly diastereoselectively (d.r. = 92:8) at 50% conversion, [9] the analogous reaction of *rac*-**1b** yielded *rac*-**5b** in a poor diastereomeric ratio (d.r. = 59:41) at comparable conversion. In sharp contrast to the results obtained in the hydrosilylation, embedding the asymmetrically substituted silicon into a cyclic framework appears to be an essential feature.

**Scheme 3:**
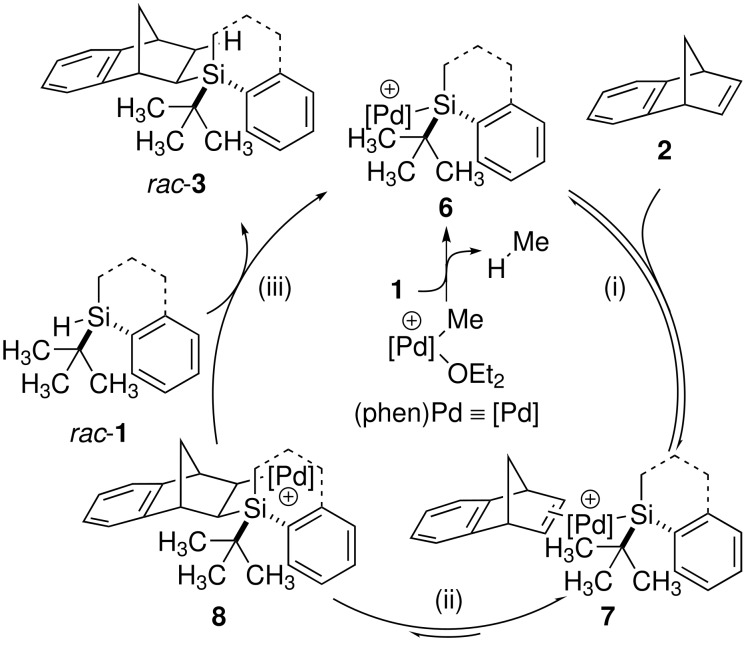
Catalytic cycle for hydrosilylation.

A comparison of the mechanisms of each reaction might serve as an explanation for this unexpected divergence. As outlined in Scheme 3, the hydrosilylation proceeds via a three-step catalytic cycle: (i) Reversible coordination of cationic silyl palladium species **6** by the alkene **2** (**6** → **7**), followed by (ii) fast and reversible migratory insertion forming β-silyl alkyl palladium intermediate **8** (**7** → **8**), and (iii) the involvement of a second silane moiety in the irreversible σ-bond metathesis. [10,16] Recent results clearly indicate step (ii) as diastereoselectivity-determining, revealing a thermodynamically controlled, reversible but highly diastereoselective migratory insertion step. [10]

**Scheme 4:**
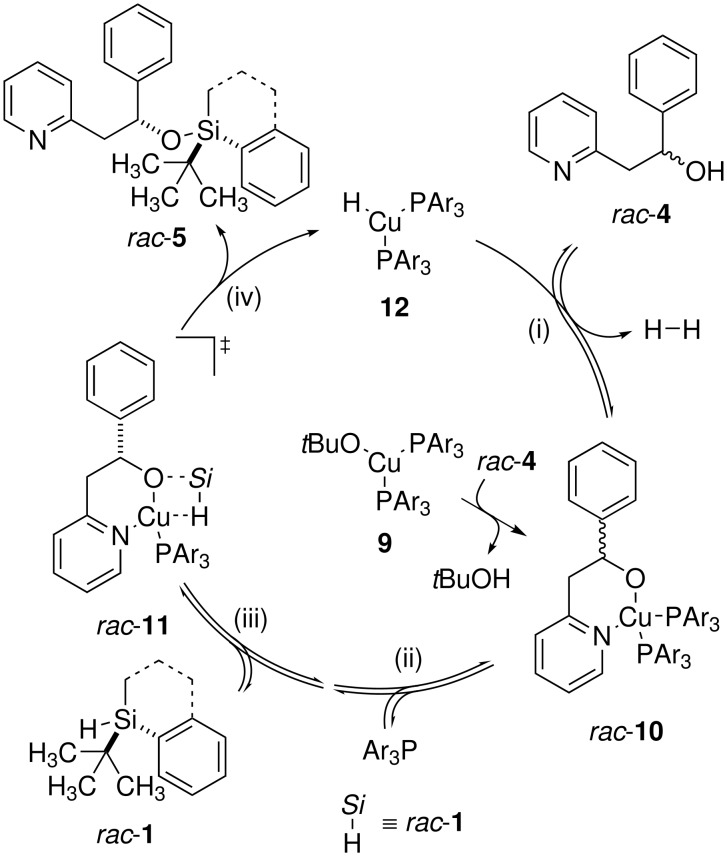
Postulated catalytic cycle for dehydrogenative coupling.

A different scenario might apply to the copper-catalyzed kinetic resolution of alcohols (Scheme 4). The phosphine-stabilized copper hydride **12** [17] is likely to be the catalytically active species, which is generated by alkoxide exchange (**9** → **10**) followed by a single catalytic turnover. The actual catalytic cycle then proceeds in a four-step propagation: (i) Coordination of pyridyl alcohol *rac*-**4** accompanied by liberation of dihydrogen (**12** → **10**), (ii) rate-limiting dissociation of one phosphine ligand to generate a free coordination site, [18] (iii) coordination of the weakly donating chiral silane (**10** → **11**), followed by (iv) an exothermic and irreversible σ-bond metathesis [19] establishing the silicon-oxygen linkage in **5** and regenerating copper hydride **12** after coordination of another phosphine ligand (**11** → **12**). With steps (ii) and (iii) being reversible and chelate **10** being capable of alkoxide exchange, that is exchange of the optical antipodes of **4**, one enantiomer of **4** is preferentially funnelled out via diastereomeric transition states (**11** → **12**).

There is one major difference between the diastereoselectivity-determining steps in these catalytic cycles: (ii) in Scheme 3 and (iv) in Scheme 4. In the migratory insertion (ii, **7** → **8**), carbon-silicon bond formation occurs between the stereogenic silicon and the prochiral carbon therefore entailing their close proximity. The newly formed stereogenic carbon is directly connected to the former source of chiral information. In contrast, the decisive asymmetrically substituted carbon atom in the alcohol substrate is more remote from the stereoselectivity-controlling silicon moiety in the silicon-oxygen bond formation (iv, **11** → **5**). The stereogenic carbon in the alcohol is not directly involved in the actual bond formation. This mechanistic picture might account for the more demanding requirements to chiral silane **1**: A cyclic framework leading to a locked conformation [11] improving the degree of organization in the stereochemistry-determining transition state **11**.

In summary, we have shown for the first time that an excellent chirality transfer from silicon to carbon is also realized with suitably substituted acyclic silanes such as **1b**. Our survey, however, underscores once more that cyclic silane **1a** is a priveleged structure and certainly generally more applicable to catalytic asymmetric processes than **1b**. The current mechanistic pictures provide a rather simple explanation for the observed stereochemical outcome of both diastereoselective carbon-silicon and silicon-oxygen bond formation. Based on this insight, further research will be devoted to the extension chiral silicon-based asymmetric catalysis.

## Supporting Information

File 1 Supporting Information. Experimental procedures and characterization data for all new compounds described in this manuscript.
