# Supplementary material for: Conformational rigidity of silicon-stereogenic silanes in asymmetric catalysis: A comparative study
Source: Beilstein J Org Chem. 2007 Feb 8;3:9. doi: 10.1186/1860-5397-3-9 (PMC1810299; doi:10.1186/1860-5397-3-9)
Supplement: File 1 — Supporting Information. Experimental procedures and characterization data for all new compounds described in this manuscript. [file Beilstein_J_Org_Chem-03-09-s001.doc]

**Conformational rigidity of silicon-stereogenic silanes in asymmetric catalysis: A comparative study**

Sebastian Rendler and Martin Oestreich*

Organisch-Chemisches Institut

Westfälische Wilhelms-Universität Münster

Corrensstrasse 40

D-48149 Münster (Germany)

Fax: (+49)251-83-36501

E-mail: martin.oestreich@uni-muenster.de

E-mail: sebastian.rendler@uni-muenster.de

*Additional File 1*

Supporting Information

**Experimental Procedures & Characterization Data**

**General Remarks**

Reagents obtained from commercial suppliers were used without further purification unless otherwise noted. **1a**^[1]^, **1b**^[2]^, **2**^[3]^, **3a**^[4,5]^, (phen)PdMe_2_^[6,7]^, [H(OEt_2_)_2_]^+^BAr_4_^−^ [Ar = 3,5-bis(trifluoromethyl)phenyl]^[8,9]^ were prepared according to known procedures. All reactions were performed in flame-dried glassware under a static pressure of argon. Liquids and solutions were transferred with syringes or double-ended needles. Solvents were dried prior to use following standard procedures (THF, CH_2_Cl_2_, toluene). Technical grade solvents for extraction or chromatography (cyclohexane, *t*-butyl methyl ether) were distilled before use. Analytical thin-layer chromatography was performed on silica gel SIL G-25 glass plates by MachereyNagel/Germany and flash chromatography on silica gel 60 (4063 μm, 230-400 mesh, ASTM) by Merck/Germany using the indicated solvents. ^1^H and ^13^C NMR spectra were recorded in CDCl_3_ on a Bruker AM 400 instrument. Mass spectra were recorded with a Finnigan MAT TSQ7000 (EI/CI-MS) or a Bruker Micro-TOF (EI/CI-MS).

(1*S**,2*R**,4*S**,Si*S**)-1-*tert*-Butyl-1-methyl-1-phenyl-1-(1,2,3,4-tetrahydro-1,4-methano-naphth-2-yl)silane (*rac*-**3b**)

A Schlenk tube was charged with a mixture of (phen)PdMe_2_ (6.7 mg, 0.021 mmol, 0.030 equiv.) and [H(OEt_2_)_2_]^+^BAr_4_^−^ [Ar = 3,5-bis(trifluoromethyl)phenyl] (21.2 mg, 0.021 mmol, 0.030 equiv.) under argon atmosphere. The solids were dissolved in anhydrous degassed CH_2_Cl_2_ (3.0 mL) at 0°C forming a pale yellow solution. At this temperature, a solution of bicyclic alkene **2** (99.5 mg, 0.700 mmol, 1.00 equiv.) and silane *rac-***1b** (137.3 mg, 0.770 mmol, 1.10 equiv.) in CH_2_Cl_2_ (4.0 mL) was added in one portion via syringe. The resulting bright yellow solution was maintained at 0°C until complete consumption of the reactants (12 h) as monitored by ^1^H NMR analysis. After addition of cyclohexane (20 mL) and a small portion of silica gel the solvents were evaporated. Purification by flash column chromatography on silica gel (cyclohexane) afforded the analytically pure product *rac*-**3b** (157 mg, 70%) as a white solid.

R*_f_* = 0.50 (cyclohexane). IR (CHCl_3_) $\tilde{\nu}$ 3068 (m), 3017 (m), 2956 (s), 2928 (s), 2881 (m), 2856 (s), 1467 (s), 1426 (m), 1252 (s), 1105 (s), 891 (m), 821 (s), 777 (s), 755 (s), 734 (s), 701 (s), 514 (s), 476 (s), 428 (s), 411 (m) cm^−1^. ^1^H NMR (400 MHz, CDCl_3_) δ 0.42 (s, 3H), 1.00 (s, 9H), 1.14 (ddd, *J* = 9.7 Hz, *J* = 7.1 Hz, *J* = 1.3 Hz, 1H), 1.40 (ddd, *J* = 13.5 Hz, *J* = 9.9 Hz, *J* = 1.8 Hz, 1H), 1.46 (ddd, *J* = 9.0 Hz, *J* = *J* = 1.5 Hz, 1H), 3.33 (br d, *J* = 1.9 Hz, 1H), 3.48 (br s, 1H), 7.06−7.16 (m, 2H), 7.18−7.24 (m, 2H), 7.28−7.38 (m, 3H), 7.42−7.51 (m, 2H) ppm. ^13^C NMR (100 MHz, CDCl_3_) δ −9.19, 18.06, 23.12, 27.80, 31.52, 44.04, 45.53, 48.29, 118.8, 121.2, 125.3, 127.4, 128.8, 135.1, 136.7, 147.1, 151.2 ppm. LR-MS (EI) m/z 320 [M^+^], 263 [(M−C_4_H_9_)^+^]. Anal. Calcd for C_22_H_28_Si: C, 82.43; H, 8.80. Found: C, 82.51; H, 8.84.

*rac*-1-Phenyl-2-(pyrid-2-yl)ethanol (*rac*-**4**)

Cerium(III) chloride (CeCl_3_·7H_2_O) (3.73 g, 10.0 mmol, 1.00 equiv.) dried by heating *in vacuo* (0.1 mbar) in an oil bath at 140°C with slow stirring for 2 h.^[10,11]^ After backfilling of the flask with argon, anhydrous THF (50 mL) was added at –78°C and stirring of the white suspension was continued for 1h. At this temperature, a solution of 2-(lithiomethyl)pyridine in THF – prepared by slow addition of *n*-BuLi (4.00 mL, 10.0 mmol, 1.0 equiv., 2.5m solution in hexanes) to a solution of 2-picoline (0.99 mL, 0.93 g, 10.0 mmol, 1.00 equiv.) in THF at –30°C and stirring for further 1 h – was added in one portion and maintained for 1 h at this temperature for further 1 h. Subsequently, benzaldehyde (1.21 mL, 1.27 g, 12.0 mmol, 1.20 equiv.) was added dropwise to the orange suspension that decolorized immediately. After 10 min at –78°C, the reaction mixture was quenched with H_2_O (40 mL) and *tert*-butyl methyl ether (20 mL) was added. The pH was adjusted to 7-8 by adding aqueous HCl (2m) and the organic phase was separated. Extraction of the aqueous phase with *tert*-butyl methyl ether (4 × 25 mL) and washing of the combined organic extracts with brine (30 mL) was followed by drying of the organic phases over Na_2_SO_4_. The solvents evaporated under reduced pressure and the residual colored solid was recrystallized from *tert*-butyl methyl ether/CH_2_Cl_2_ to give the title compound *rac*-**4** (1.47 g, 74%) as a white crystalline solid.

R*_f_* = 0.12 (cyclohexane/*tert*-butyl methyl ether 50:50). mp. 107°C. IR (CHCl_3_) $\tilde{\nu}$ 3308 (s), 3089 (s), 3066 (s), 3031 (s), 2906 (m), 2247 (s),1952 (w), 1882 (w), 1760 (vw), 1597 (s), 1570 (s), 1495 (m), 1475 (s), 1453 (s), 1438 (s), 1338 (m), 1279 (m), 1280 (m), 1150 (w), 1100 (m), 1078 (w), 1054 (m), 1028 (m), 1019 (m), 1001 (w), 903 (m), 816 (w), 735 (w), 607 (w), 577 (m), 546 (m) cm^−1^. ^1^H NMR (400 MHz, CDCl_3_) δ 3.08−3.19 (m, 2H), 5.17 (dd, *J* = 8.3 Hz, *J* = 3.9 Hz, 1H), 5.69 (br s, 1H), 7.11 (br d, *J* = 8.0 Hz, 1H), 7.18 (ddd, *J* = 7.6 Hz, *J* = 4.9 Hz, *J* = 1.1 Hz, 1H), 7.26 (m_c_, 1H), 7.35 (m_c_, 1H), 7.43 (m_c_, 1H), 7.43 (m_c_, 1H), 7.61 (ddd, *J* = *J* = 7.6 Hz, *J* = 1.8 Hz, 1H), 8.54 (ddd, *J* = 4.9 Hz, *J* = 1.6 Hz, *J* = 1.0 Hz, 1H) ppm. ^13^C NMR (100 MHz, CDCl_3_) δ 45.76, 73.40, 121.8, 123.9, 125.9, 127.3, 128.4, 136.9, 144.2, 148.7, 159.9 ppm. LR-MS (CI/NH_3_) m/z 200 [(M+H)^+^]. Anal. Calcd for C_13_H_13_NO: C, 78.36; H, 6.58; N, 7.03. Found: C, 78.13; H, 6.63; N, 6.95.

(Si*S**,*S**)-2-[2-(1-*tert*-Butyl-1,2,3,4-tetrahydro-1-silanaphthyloxy]-2-phenylethyl]pyridine (*rac*-**5a**)^[12]^

A Schlenk tube was charged with CuCl (2.0 mg, 0.020 mmol, 0.050 equiv.) and (xylyl)_3_P (13.9 mg, 0.040 mmol, 0.100 equiv.) under argon atmosphere. The solids were suspended in anhydrous, degassed toluene (1.0 mL) at room temperature. To the stirred suspension, NaO*t*Bu (1.9 mg, 0.020 mmol. 0.050 equiv.) was added and subsequently stirred for 2 min upon which a clear, pale yellow solution had formed. At room temperature, a solution of pyridyl alcohol *rac*-**4** (79.7 mg, 0.400 mmol, 1.00 equiv.) and silane *rac*-**1a** (45.0 mg, 0.220 mmol, 0.550 equiv.) in toluene (3.0 mL) was added via syringe. The resulting bright yellow solution was maintained at room temperature for 8 h until complete conversion of silane was detected by ^1^H NMR analysis. After addition of cyclohexane (10 mL) and a small portion of silica gel the solvents were evaporated. Purification by flash column chromatography on silica gel with cyclohexane/*tert*-butyl methyl ether 94:6 → 50:50) afforded ether *rac*-**5a** (79.5 mg, 99%, d.r. = 92:8 by ^1^H NMR) as a viscous oil and as the unreacted alcohol *rac*-**4** (39.5 mg, 99%) as a white solid.

R*_f_* = 0.15 (cyclohexane/*tert*-butyl methyl ether 90:10). IR (CHCl_3_) $\tilde{\nu}$ 3060 (w), 3031 (w), 3004(w), 2953 (m), 2929 (s), 2891 (m), 2857 (s), 2254 (m), 2212 (m), 1951 (w), 1593 (s), 1570 (m), 1493 (w), 1474 (s), 1463 (m), 1453 (m), 1437 (s), 1402 (w), 1362 (m), 1293 (m), 1269 (m), 1206 (m), 1143 (m), 1128 (m), 1078 (s), 1068 (s), 1012 (m), 975 (m), 843 (m), 825 (s), 797 (s), 681 (m), 606 (m), 579 (m), 546 (m), 509 (w) cm^−1^. LR-MS (CI/NH_3_) m/z 402 [(M+H)^+^]. Anal. Calcd for C_26_H_31_NOSi: C, 77.76; H, 7.78; N, 3.49. Found: C, 77.47; H, 8.06; N, 3.28.

NMR data for (Si*S**,*S**)-5a^[13]^ (major): ^1^H NMR (400 MHz, CDCl_3_) δ 0.33 (dddd, *J* = 15.0 Hz, *J* = 6.2 Hz, *J* = 3.9 Hz, *J* = 1.2 Hz, 1H), 0.56 (ddd, *J* = 15.1 Hz, *J* = 12.1 Hz, *J* = 5.2 Hz, 1H), 0.84 (s, 9H), 1.15–1.29 (m, 1H), 1.70 (m_c_, 1H), 2.39 (ddd, *J* = 16.0 Hz, *J* = 11.0 Hz, *J* = 2.7, 1H), 2.52 (br dd, *J* = 15.9 Hz, *J* = 5.6 Hz, 1H), 3.00 (dd, *J* = 13.2 Hz, *J* = 3.8 Hz, 1H), 3.12 (dd, *J* = 13.0 Hz, *J* = 9.6 Hz, 1H), 4.96 (dd, *J* = 9.6 Hz, *J* = 3.2 Hz, 1H), 6.72 (dd, *J* = 7.3 Hz, *J* = 1.5 Hz, 1H), 6.92–7.05 (m, 2H), 7.09–7.37 (m, 8H), 7.57 (ddd, *J* = *J* = 7.5 Hz, *J* = 1.2 Hz, 1H), 8.50 (br d, *J* = 4.4 Hz, 1H) ppm. ^13^C NMR (100 MHz, CDCl_3_) δ 9.68, 18.63, 22.61, 26.07, 50.18, 75.59, 121.4, 125.0, 125.3, 126.2, 127.3, 128.2, 128.3, 129.1, 129.3, 130.5, 135.0, 135.6, 136.0, 145.2, 149.2, 150.6, 159.1 ppm.

NMR data for (Si*S**,*R**)-5a (minor): ^1^H NMR (400 MHz, CDCl_3_) δ 0.19 (dddd, *J* = 15.1 Hz, *J* = 6.2 Hz, *J* = 4.0 Hz, *J* = 1.5 Hz, 1H), 0.54 (ddd, *J* = 15.0 Hz, *J* = 12.0 Hz, *J* = 5.3 Hz, 1H), 0.81 (s, 9H), 1.34–1.48 (m, 1H), 1.81 (m_c_, 1H), 2.42 (m_c_, 1H), 2.61 (m, 1H), 3.05 (dd, *J* = 13.2 Hz, *J* = 4.3 Hz, 1H), 3.12 (dd, *J* = 13.0 Hz, *J* = 9.6 Hz, 1H), 5.05 (dd, *J* = 9.0 Hz, *J* = 4.0 Hz, 1H), 6.84 (dd, *J* = *J* = 7.3 Hz, 1H), 6.92−7.05 (m, 2H), 7.09−7.37 (m, 8H), 7.64 (dd, *J* = 7.5 Hz, *J* = 1.6 Hz, 1H), 8.55 (br d, *J* = 5.0 Hz, 1H) ppm. ^13^C NMR (100 MHz, CDCl_3_) δ 9.39, 18.71, 23.02, 26.06, 35.63, 50.18, 75.96, 121.5, 124.6, 125.3, 126.2, 127.1, 128.0, 128.2, 128.6, 129.7, 130.9, 134.6, 135.6, 136.0, 144.9, 149.2, 150.2, 159.1 ppm.

(Si*S**,*S**)-2-[2-(1-*tert*-Butyl-1-methyl-1-phenylsilyloxy)-2-phenylethyl]pyridine (*rac*-**5b**)

A Schlenk tube was charged with CuCl (5.0 mg, 0.050 mmol, 0.050 equiv.) and (xylyl)_3_P (34.6 mg, 0.0100 mmol, 0.100 equiv.) under argon atmosphere. The solids were suspended in anhydrous, degassed toluene (2.5 mL) at room temperature. To the stirred suspension, NaO*t*Bu (4.8 mg, 0.050 mmol. 0.050 equiv.) was added and subsequently stirred for 2 min upon which a clear, pale yellow solution had formed. At room temperature, a solution of pyridyl alcohol *rac*-**4** (199.3 mg, 1.000 mmol, 1.000 equiv.) and silane *rac*-**1b** (98.1 mg, 0.550 mmol, 0.550 equiv.) in toluene (7.5 mL) was added via syringe. The resultig bright yellow solution was mainainted at room temperature for 8 h until complete conversion of silane was indicated by ^1^H NMR analysis. After addition of cyclohexane (10 mL) and a small portion of silica gel the solvents were evaporated. Purification by flash column chromatography on silica gel with cyclohexane/*tert*-butyl methyl ether 94:6 → 50:50) afforded first silyl ether *rac*-**5b** (192 mg, 98%, d.r. = 59:41 by ^1^H NMR) as a viscous oil and as a second fraction the remaining alcohol *rac*-**4** (67 mg, 70%) as a white solid.

R*_f_* = 0.15 (cyclohexane/*tert*-butyl methyl ether 85:15). IR (CHCl_3_) $\tilde{\nu}$ 3069 (w), 3019 (w), 2958 (s), 2928 (m), 2856 (m), 2360 (w), 1647 (s), 1468 (s), 1426 (m), 1361 (w), 1254 (s), 1105 (s), 752 (s), 735 (s), 701 (m), 463 (s), 412 (s) cm^−1^. LR-MS (CI/MeOH) m/z 376 [(M^+^+H)]. Anal. Calcd for C_24_H_29_NOSi: C, 76.75; H, 7.78; N, 3.73. Found: C, 76.63; H, 7.76; N, 3.50.

NMR data for (Si*S**,*S**)-5b (major)^[14]^: ^1^H NMR (400 MHz, CDCl_3_) δ −0.04 (s, 3H), 0.81 (s, 9H), 3.14 (dd, *J* = 12.8 Hz, *J* = 4.1 Hz, 1H), 3.24 (dd, *J* = 12.6 Hz, *J* = 8.8 Hz, 1H), 5.21 (dd, *J* = 9.0 Hz, *J* = 4.2 Hz, 1H), 7.10–7.42 (m, 11H), 7.59 (ddd, *J* = *J* = 7.8 Hz, *J* = 1.8 Hz, 1H), 8.55−8.63 (m, 1H) ppm. ^13^C NMR (125 MHz, CDCl_3_) δ −7.14, 18.62, 25.99, 50.17, 75.96, 125.2, 126.1, 127.1, 127.37, 128.1, 129.1, 134.7, 135.7, 136.1, 144.9, 149.2, 158.9 ppm.

NMR data for (Si*S**,*R**)-5a (minor): ^1^H NMR (500 MHz, CDCl_3_) δ −0.10 (s, 3H), 0.82 (s, 9H), 3.12 (dd, *J* = 12.9 Hz, *J* = 3.7 Hz, 1H), 3.23 (dd, *J* = 13.0 Hz, *J* = 8.9 Hz, 1H), 5.14 (dd, *J* = 9.0 Hz, *J* = 4.2 Hz, 1H), 7.10−7.42 (m, 11H), 7.54 (ddd, *J* = *J* = 7.6 Hz, *J* = 2.0 Hz, 1H), 8.55−8.63 (m, 1H). ppm. ^13^C NMR (100 MHz, CDCl_3_) δ −6.76, 18.48, 25.96, 50.21, 75.78, 121.4, 125.3, 126.2, 127.1, 127.3, 128.2, 129.2, 134.5, 135.6, 136.1, 145.0, 149.1, 158.9 ppm.

**References**

[1] Rendler S, Auer G, Keller M, Oestreich M: *Adv Synth Catal* 2006, **348**:1171–1182.

[2] Larson GL, Torres E: *J Organomet Chem* 1985, **293**:19–27.

[3] Wittig G, Knauss E: *Chem Ber* 1958, **91**:895–905.

[4] Oestreich M, Rendler S: *Angew Chem Int Ed* 2005, **44**:1661−1664.

[5] Rendler S, Oestreich M, Butts CP, Lloyd-Jones GC: *J Am Chem Soc* 2007, **129**: 502−503.

[6] De Graaf W, Boersma J, Smeets WJJ, Spek AL, Van Koten G: *Organometallics* 1989, **8**:2907–2917.

[7] Rix FC, Brookhart M, White PS: *J Am Chem Soc* 1996, **118**:2436–2448.

[8] Brookhart M, Grant B, Volpe Jr AF: *Organometallics* 1992, **11**: 3920–3922.

[9] Yakelis NA, Bergman RG: *Organometallics* 2005, **24**:3579–3581.

[10] Imamoto T, Sugiura Y, Takaiyama N: *Tetrahedron Lett* 1984, **25**:4233–4236.

[11] Imamoto T, Kusumoto T, Tawarayama Y, Sugiura Y, Yasushi M, Takeshi H, Hatanaka Y, Yokoyama M: *J Org Chem* 1984, **49**:3904–3912.

[12] Rendler S, Auer G, Oestreich M: *Angew Chem Int. Ed.* 2005, **44**:7620−7624.

[13] Assignment of relative configuration based on the reaction of enantioenriched silane **1a** with known absolute configuration as in ref. ^[12]^ and by comparison of the optical rotation of the remaining enantioenriched alcohol **4** with literature data given in ref. ^[15]^.

[14] Assignment of relative configuration of the major diastereomer analogously to **5a**.

[15] Yu C, Meth-Cohn O: *Tetrahedron Lett* 1999, **40**: 6665–6668.
